# A Network Pharmacology Study on the Active Components and Targets of the Radix Ginseng and Radix Bupleuri Herb Pair for Treating Nonalcoholic Fatty Liver Disease

**DOI:** 10.1155/2022/1638740

**Published:** 2022-02-08

**Authors:** Qiang Zhang, Li Zhang, Kaili Liu, Haonan Shang, Jun Ruan, Zhonghai Yu, Shengxi Meng, Fang Liang, Tianzhan Wang, Hongyan Zhang, Wenbo Peng, Yuxin Wang, Junming Chen, Tiegang Xiao, Bing Wang

**Affiliations:** ^1^Department of Traditional Chinese Medicine, Shanghai Sixth People's Hospital Affiliated to Shanghai Jiao Tong University, Shanghai, China; ^2^Department of Traditional Chinese Medicine, Shanghai Pudong New District Zhoupu Hospital, Shanghai University of Medicine & Health Sciences, Shanghai, China; ^3^Department of Gastroenterology, Shanghai Traditional Chinese Medicine-Integrated Hospital, Shanghai University of Traditional Chinese Medicine, Shanghai, China

## Abstract

**Objective:**

To explore the potential active components and corresponding target herb pairs of Radix Ginseng (Renshen) and Radix Bupleuri (Chaihu) in the treatment of nonalcoholic fatty liver disease (NAFLD) through network pharmacology and in vitro experiments.

**Methods:**

The active components and potential targets of the herb pair of Renshen and Chaihu were screened through a network database system, and Venn analysis was performed with the obtained NAFLD targets. The intersecting targets were analysed for gene ontology (GO) functions and Kyoto Encyclopedia of Genes and Genome (KEGG) pathways, and a protein-protein interaction (PPI) network was generated. Cytoscape software was used to construct active component-target networks of the Renshen and Chaihu herb pair. Free fatty acids were added to the HepG2 cell line to create high-fat models that were treated with different concentrations of stigmasterol. The effect of stigmasterol on the lipid metabolism in HepG2 cells and PPAR*γ*-knockdown cells was determined by oil red O staining, Nile red staining, and TG level. PPAR*γ* and UCP-1 mRNA, and protein expression levels were detected by qRT-PCR and Western blot analyses, respectively.

**Results:**

Twenty active components obtained from the Renshen and Chaihu herb pair were identified. The herb pair active component-target network showed that both Renshen and Chaihu contained stigmasterol and kaempferol as active components. The PPI network comprised 63 protein nodes. GO enrichment analysis and KEGG pathway enrichment analysis showed that the targets were mainly involved in lipid metabolism. Eight core targets were identified: AKT1, PPARG, MAPK3, TNF, TP53, SIRT1, STAT3, and PPARA. In vitro experiments demonstrated that stigmasterol reduced lipid accumulation and TG levels in HepG2 cells, and the mechanism may have been related to the activation of the PPAR*γ*-UCP-1 signalling pathway.

**Conclusion:**

This study preliminarily illustrated the potential components and corresponding core targets of the Renshen and Chaihu herb pair in treating NAFLD. The effect of stigmasterol on the PPAR*γ*-UCP-1 signalling pathway in enhancing lipid metabolism may represent one of the mechanisms of the Renshen and Chaihu herb pair in the treatment of NAFLD. The results provide new evidence and research insights to reveal the roles of Renshen and Chaihu in the management of NAFLD.

## 1. Introduction

Nonalcoholic fatty liver disease (NAFLD) is caused by metabolic stress-induced liver injury closely related to insulin resistance and genetic susceptibility. One of the most common hepatic diseases worldwide, NAFLD is a spectrum disease that includes nonalcoholic fatty liver (NAFL), nonalcoholic steatohepatitis (NASH), and liver cirrhosis [1]. A recent systematic review has indicated that the prevalence of NAFLD is as high as 29.2% in China [2]. The pathophysiology of NAFLD is not yet clear; however, lipotoxic response is clearly a central mechanism of NAFLD development and progression [3]. Based on this understanding, various chemical agents have been developed and are under investigation to explore their potential benefits [4]. However, no drugs specific for NAFLD have been approved to date [5, 6]. In China, many patients seek medical consultations from practitioners of traditional Chinese medicine (TCM) and consume Chinese herbal medicines (CHMs).

Various studies have found the potential therapeutic effect of CHMs on NAFLD [7–9]. The Radix Ginseng (Renshen) and Radix Bupleuri (Chaihu) herb pair is a commonly prescribed herb combination. Nevertheless, the specific active components are unknown. In addition, due to the complexity of active components in Renshen and Chaihu, other underlying mechanisms may confer protection against NAFLD. These issues deserve further investigation.

Network pharmacology, characterized by the comprehensive presentation of herb-target-disease networks, may be a valuable approach to explore the outstanding issues regarding the herb pair effect on NAFLD [10, 11]. Network pharmacology can be used to identify the potential active components and corresponding therapeutic targets of the Renshen and Chaihu herb pair for treating NAFLD. Additional precise identifications can then be made to verify their effects.

In this study, we first used the network database to identify the active components of herbs similar to the Renshen and Chaihu herb pair and employed SwissTargetPrediction to predict the potential therapeutic targets. Then, an intersection analysis of potential targets of the herb pair and known therapeutic targets of NAFLD was performed to identify the core targets. In addition, a PPI analysis, GO enrichment analysis, and KEGG pathway enrichment analysis were performed, and an active component-target network was established. Finally, in vitro experiments verified that the Renshen and Chaihu herb pair had a pharmacodynamic effect on the components of stigmasterol by activating the PPAR*γ* signalling pathway, clarified the material basis of the Renshen and Chaihu herb pair for the treatment of NAFLD, and provided an experimental basis for the treatment of NAFLD.

## 2. Methods

### 2.1. Data Preparation

A total of 39 active components in the herb pair were manually extracted from the TCM System Pharmacology Database (TCMSP, http://lsp.nwu.edu.cn/tcmsp.php, version 2.3) [12]. Relationships among drug components, targets, and diseases were obtained from the TCMSP; this database contains the pharmacokinetic properties of a variety of Chinese medicines, including their oral bioavailability, similar herbs, and intestinal epithelial permeability.

### 2.2. Active Component Prediction

After active component screening, an absorption, distribution, metabolism, and excretion (ADME) evaluation system was used to select potential active components. We selected two pharmacokinetic parameters, namely, oral bioavailability (OB) [13] and herb similarity (DL) [14], to identify the active components of the herb pair. In this study, OB ≥ 30% and DL ≥ 0.18 were used as the screening criteria for active components. Active components that met the criteria were regarded as candidate components for subsequent analysis.

### 2.3. Target Fishing

To obtain the 2D structure of active components, we evaluated chemical data in PubChem (https://pubchem.ncbi.nlm.nih.gov) and saved the 2D structure in sdf format. PubChem is the largest freely accessible database of chemical information in the world, providing chemical properties, physical properties, biological activity, safety, and toxicity information of chemical substances. The 2D structure of the active components was sequentially imported into SwissTargetPrediction (http://www.swisstargetprediction.ch) [15], which was used to predict the most likely macromolecular target based on the 2D or 3D structure of the biologically active small molecules. We selected *Homo sapiens* as the species to predict the potential targets of Renshen and Chaihu.

### 2.4. The Disease Target of NAFLD

NAFLD-related genes were obtained from the DisGeNET database (https://www.disgenet.org) [16]. The DisGeNET database has publicly available genes and variants related to human diseases. We used the key term “non-alcoholic fatty liver disease” to search, and we obtained 333 genes. We then performed a Venn diagram analysis of the intersection of the active components and NAFLD genes.

### 2.5. Protein-Protein Interaction Data

The PPI data of 63 intersection targets of the herb pair and NAFLD were extracted from STRING (http://string-db.org/) [17], and the parameters were filtered using *Homo sapiens* (confidence level >0.4). The STRING database is frequently used to generate PPI networks and mine core regulatory genes.

### 2.6. GO Enrichment Analysis and KEGG Pathway Enrichment Analysis

The intersection target of NAFLD and the herb pair was analysed through gene ontology (GO) analysis and Kyoto Encyclopedia of Genes and Genomes (KEGG) pathway analysis in the Metascape database (https://metascape.org/) [18]. An adjusted *P* value <0.05 was set as the standard, and the species was set to *Homo sapiens*. The biological processes and pathways related to NAFLD were obtained.

### 2.7. Topological Analysis of the Active Component-Target Network

Topological analysis was performed by importing TSV format files from STRING to Cytoscape 3.7.02 software. Cytoscape was used to visualize biological pathway networks and molecular interaction networks. First, we calculated the topology parameters of the PPI network through the NetworkAnalyser tool. Then, using the degree parameter, we sorted the target area from large to small and the colour from light to dark. Using the Generate Style in the Statistics tool, we sorted the lines between the targets from thick to thin according to the combined score, and we sorted the colours from light to dark. On the basis of results greater than the median of the twofold degree value of the PPI network, we identified the core targets for the regulation of NAFLD by active components. Finally, we obtained 8 core targets and matched them with the active components. Thus, we identified 20 active components related to the 8 core targets from the intersection targets.

### 2.8. Network Construction

An active component-target network was established. The core target and its matching active components were obtained on the strength of PPI data and topological analysis. Then, the network of herb pair active component-core target of NAFLD was constructed.

### 2.9. Establishment of the NAFLD Cell Model

The AML12 normal mouse liver cell line and HepG2 human liver cancer cell line were donated by the Institute of Digestive Diseases of Shanghai University of Traditional Chinese Medicine. Both cell lines were seeded in 6-well plates and subcultured in the laboratory in DMEM containing 10% foetal bovine serum and two antibiotics at 37°C in 95% air humidity and 5% CO_2_ and maintained in good condition. Passaged cells were digested with 0.25% trypsin.

### 2.10. Experiment Grouping and Modelling

When reaching 60%–70% confluency, the cells were randomly divided into a blank control group (C), model group (M), low-dose stigmasterol group (L), and high-dose stigmasterol group (H). Group M was supplemented with 400 *μ*M oleic acid and 200 *μ*M palmitic acid. In addition to free fatty acids, the L and H groups were stimulated with low-dose (30 *μ*M) and high-dose (60 *μ*M) stigmasterol, respectively. The cells were treated for 24 hours, and each group was set up with 3 replicate wells. The cells were incubated in an incubator at 37°C with 5% CO_2_ and saturating humidity.

### 2.11. Efficacy Testing

Oil red O staining and Nile red staining were used to observe cell changes and the number of lipid droplets under a microscope to determine the pathological changes of the cells. A kit was used to detect biochemical indicators, including TG levels. RT-PCR was performed to detect the mRNA expression levels of PPAR*γ*, PPAR*α*, CPT-2, and UCP-1. Western blot analysis was performed to detect the protein expression levels of PPAR*γ*, PPAR*α*, CPT-2, and UCP-1. Immunofluorescence technology was used to detect PPAR*γ* and PPAR*α*.

### 2.12. Generation of PPAR*γ*-Knockdown Cell Lines

CRISPR/Cas9 technology was used to construct PPAR*γ*-knockdown cell lines. Resuspended 293T cells were seeded in a culture dish. Transfection reagent (30 *μ*l) and Opti-MEM (100 *μ*l) medium were then mixed in a 1.5 EP tube and incubated at room temperature for 5 minutes. PMG2D, PSPAX2, and the target plasmid were mixed with 100 *μ*l of the Opti-MEM medium in a 1.5 EP tube at a ratio of 1 : 3:4 (total of 2.5 *μ*g) and incubated at room temperature for 5 minutes. The solutions were then mixed together and incubated at room temperature for 20 minutes. The medium (10 ml) was added to the culture plate followed by the addition of the mixture, and the medium was changed after 24 hours of cultivation. The virus was harvested after 48 hours of cultivation. The original medium was removed from the HepG2 cells, and the virus was added to the culture dish. In addition, we added 1% polybrene to enhance the infection efficiency. After 24 hours of infection, the normal medium was changed, and the screening marker was added 48 hours later. After stable proliferation, a cell line expressing knocked down PPAR*γ* was obtained.

### 2.13. Statistical Analysis

After performing a uniformity test of variance, the results were analysed by one-way analysis of variance (ANOVA) for comparison. *P* < 0.05 was considered statistically significant.

## 3. Results

### 3.1. Network Database Analysis of the Effective Material Basis of the Renshen and Chaihu Herb Pair

**Figure 1:**
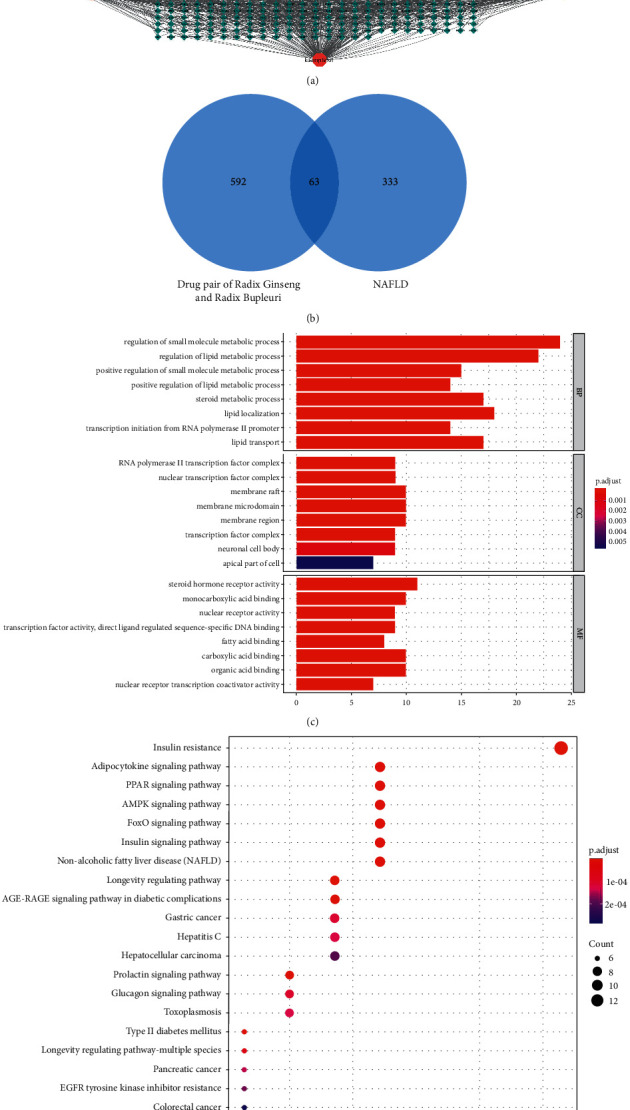
Network database analysis of the effective material basis of the Renshen and Chaihu herb pair. (a) Target biological network of the Renshen and Chaihu herb pair. (b) Venn analysis of the Renshen and Chaihu herb pair and NAFLD targets. (c) GO enrichment analysis of intersecting targets. (d) KEGG pathway analysis of intersecting targets.

According to the defined value of bioavailability, herb-like properties, and deduplication processing, 17 active components contained in Chaihu (Table 1) and 22 active components contained in Renshen (Table 2) were obtained from the TCMSP database. Targets of the active components were predicted. After removing duplications, 592 targets were obtained. Renshen and Chaihu herb pair active component-potential target regulatory networks were constructed using Cytoscape 3.7.2 software (Figure 1(a)). The 592 targets of the Renshen and Chaihu herb pair were crossed with the 333 NAFLD-related targets obtained from the DisGeNET database. In total, 63 crossed targets were screened, including HSD11B1, CNR2, PTPN1, CYP17A1, NR1H4, GPR119, FABP4, PPARD, APP, TEK, TNF, ALDH2, FDFT1, NPC1L1, NR1H3, SREBF2, CYP2C19, PPARA, VDR, PPARG, ALOX15, F2, ALOX5, GSK3B, MMP13, AKT1, INSR, TERT, ESRRA, MTOR, ITK, ADAM17, JAK2, CASP1, DPP4, REN, SIRT1, RBP4, GCK, MMP1, CPT1A, TRPV1, NAMPT, STAT3, FABP5, FABP1, MAPK3, PRKCE TP53, FFAR4, NR0B2 AGTR1, HNF4A, S1PR1, NQO1, SHH, NR1I2, CDK8, NR3C2, CCNC, TGFB1, PRKAB1, and PRKAA2 (Figure 1(b)).

The 63 overlapping target genes were subjected to GO enrichment analysis and KEGG pathway enrichment analysis. The GO enrichment analysis was performed to identify target genes involved in the biological process (BP), cellular component (CC), and molecular function (MF) categories. The top 8 terms were used to create a bar graph according to adjusted *P* value rankings (Supplemental Table 1, Figure 1(c)). The genes in the biological process category are involved in the regulation of small-molecule metabolic processes, regulation of lipid metabolic processes, positive regulation of small-molecule metabolic processes, positive regulation of lipid metabolic processes, steroid metabolic processes, lipid localization, transcription initiation from the RNA polymerase II promoter, and lipid transport. In the cellular component category, the genes are involved in the RNA polymerase II transcription factor complex, nuclear transcription factor complex, membrane rafts, membrane microdomains, membrane regions, transcription factor complexes, neuronal cell bodies, and the apical side of cells. In the molecular function category, the genes are involved in steroid hormone receptor activity, monocarboxylic acid binding, nuclear receptor activity, transcription factor activity, direct ligand-regulated sequence-specific DNA binding, fatty acid binding, carboxylic acid binding, organic acid binding, and nuclear receptor transcription coactivator activity. KEGG pathway enrichment analysis revealed 118 related pathways. A bubble chart was generated according to the *P* values and ratios of related genes (Supplemental Table 2, Figure 1(d)). The bubble chart shows the top 20 pathways, which mainly involved insulin resistance, the adipocytokine signalling pathway, and the PPAR signalling pathway.

### 3.2. Network Database System Analysis of the Potential Targets of Renshen and Chaihu Herb Pairs

**Figure 2:**
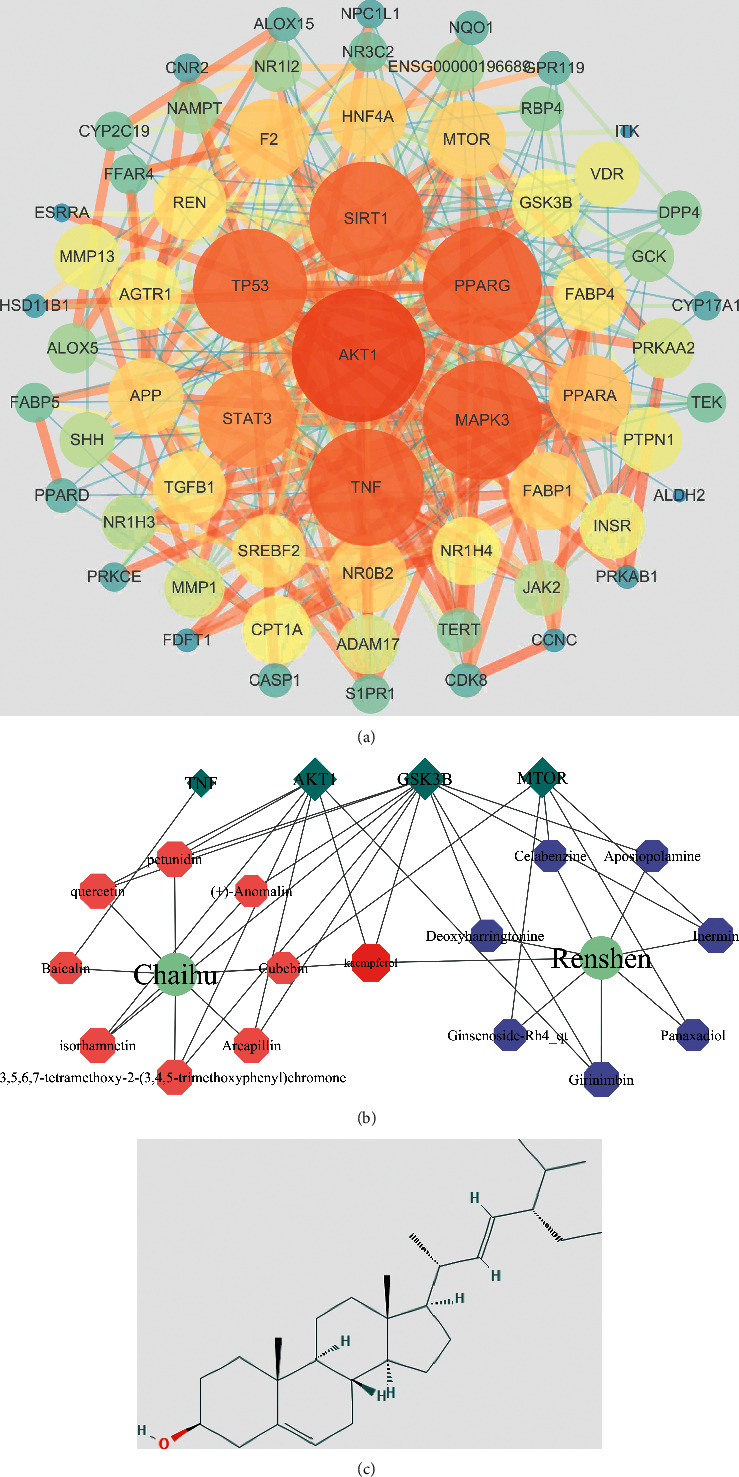
Network database system analysis of the potential targets of the Renshen and Chaihu herb pair. (a) Construction and analysis of the PPI network of intersecting targets. (b) Renshen and Chaihu herb pair component and core target regulatory network. (c) Molecular structure of stigmasterol.

The STRING database was used to establish a PPI network of the intersecting targets of the Renshen and Chaihu herb pair and NAFLD, which involved 63 protein nodes and 351 connections. Topological analysis showed that the median of the twofold degree value of the network was 18, and 8 targets, AKT1, PPARG, MAPK3, TNF, TP53, SIRT1, STAT3, and PPARA, had degree values greater than or equal to 18 (Figure 2(a)). The median of the twofold degree value of this PPI network was 18, which conformed to the requirements of the 8 targets: AKT1, PPARG, MAPK3, TNF, TP53, SIRT1, STAT3, and PPARA. On the basis of the herb pair active component-potential target network, we then determined whether the active components acted on these targets and constructed a regulatory network of components and core targets using Cytoscape 3.7.2 software to visualize any relationship (Figure 2(b)). The network contained 30 nodes and 48 connections, and 28 pairs of connections were found for the components of the Renshen and Chaihu herb pair and the targets.

The 3D structures of AKT1, PPARG, MAPK3, TNF, TP53, SIRT1, STAT3, and PPARA were obtained from the PDB database, and the 3D structure of each monomer component in mol2 format was obtained from the ZINC and TCMSP databases. We processed ligands and receptors with hydrogenation and calculated the charge and other steps for use in AutoDock Vina software of semiflexible docking to identify the interaction between the ligands and protein amino acid residues of the proteins. Lower binding energies indicated better docking (Table 3). According to the results of the molecular docking analysis and related literature, we focused on the relationship between stigmasterol and PPAR*γ* (Figure 2(c)).

### 3.3. Stigmasterol Reduces Lipid Accumulation in Cells Treated with Free Fatty Acids

**Figure 3:**
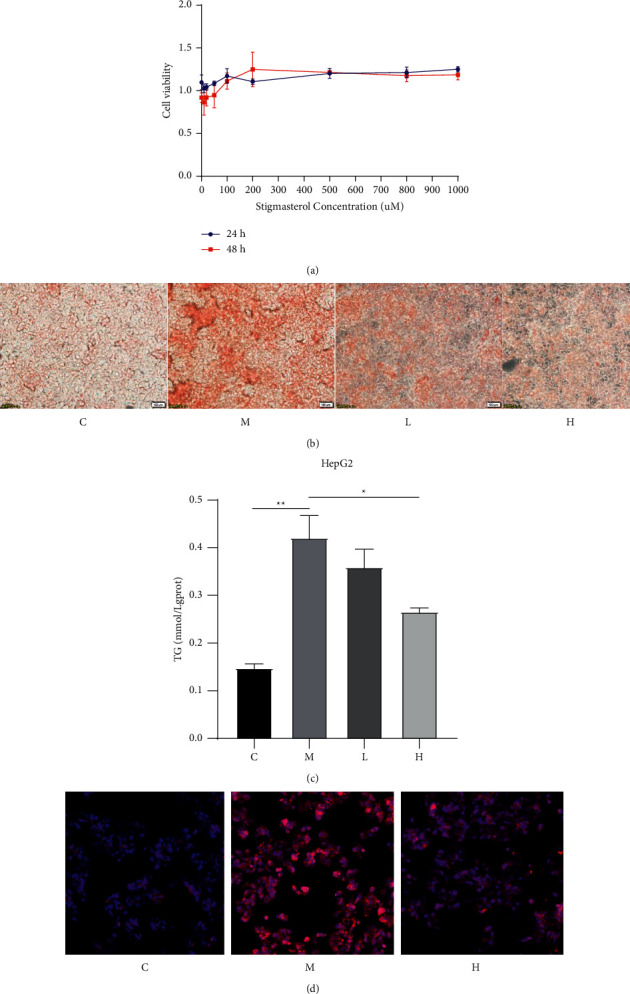
Stigmasterol reduces lipid accumulation in cells treated with free fatty acids. Cells were treated as described in the “Methods” section. After treatment, cells were harvested. (a) Cytotoxicity was detected by a WST-1 kit. (b) A total of 400 *μ*M oleic acid, 200 *μ*M palmitic acid, or 30 *μ*M/60 *μ*M stigmasterol was used to treat AML12 cells and HepG2 cells, respectively. Oil Red O test to detect the degree of cell fat change. (c) The TG level was detected by a kit and then normalized, as shown in the bar graph. (d) Nile red staining was imaged using a laser confocal microscope. ^*∗*^*P* < 0.05 and ^*∗∗*^*P* < 0.01 compared to group C ^*∗*^*P* < 0.05 and ^*∗∗*^*P* < 0.01 compared to group M.

To rule out the cytotoxicity of stigmasterol to HepG2 cells, 0–1000 *μ*M stigmasterol was used to treat HepG2 cells for 24 hours and 48 hours, and the results showed no obvious cytotoxicity (Figure 3(a)). HepG2 cells were treated with different concentrations of stigmasterol for 24 hours, stained with Oil Red O and imaged using a microscope. Compared with group C, more coloured lipid droplets were deposited in the cells of group M, and the rate of lipid change was significantly higher in group M than in group C. The number of lipid droplets was decreased after stigmasterol treatment (Figure 3(b)). Compared with group C, the TG content increased in group M (*P* < 0.01). After low-dose stigmasterol intervention, the TG content did not significantly change (*P* > 0.05). After high-dose stigmasterol intervention, the TG content significantly decreased, and the difference was statistically significant (*P* < 0.01) (Figure 3(c)). Compared to group C, Nile red staining of HepG2 cells showed that the lipid content in group M was significantly increased and that stigmasterol treatment was significantly more effective in group M (Figure 3(d)).

### 3.4. Stigmasterol Activates the Expression Level of PPAR*γ* and Downstream UCP-1

**Figure 4:**
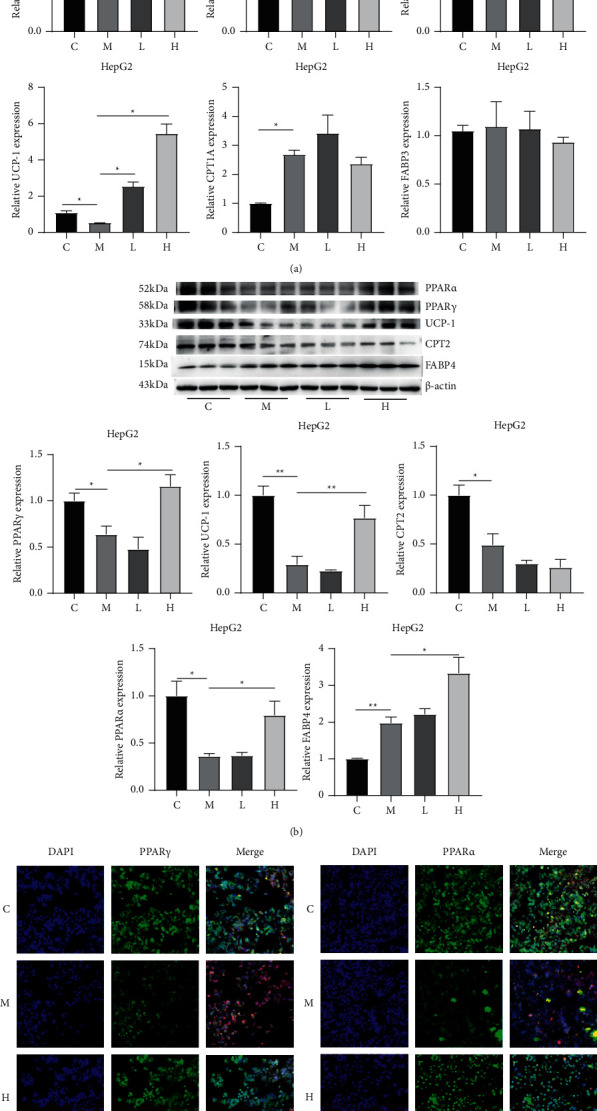
Stigmasterol activates PPAR*γ* and its downstream targets. Cells were treated as described in the “Methods” section. After treatment, the cells were harvested. (a) RT-PCR was performed to detect the mRNA expression levels of PPAR*γ*, CPT-2, and UCP-1. (b) Western blot analysis was performed to detect the protein expression levels of PPAR*γ*, CPT-2, and UCP-1. (c) Immunofluorescence was used to detect the protein level of PPAR*γ* and PPAR*α*. ^*∗*^*P* < 0.05 and ^*∗∗*^*P* < 0.01 compared to group C ^*∗*^*P* < 0.05 and ^*∗∗*^*P* < 0.01 compared to group M.

RT-PCR showed that compared to those in group C, the mRNA levels of PPAR*γ*, CPT-2, and UCP-1 in group M were significantly reduced (*P* < 0.05). After treatment with low-dose stigmasterol and high-dose stigmasterol, the mRNA levels of PPAR*γ*, CPT-2, and UCP-1 were significantly increased (*P* < 0.05) (Figure 4(a)). The WB analysis showed that compared to those in group C, the protein levels of PPAR*γ*, CPT-2, and UCP-1 in group M were significantly reduced (*P* < 0.05). After treatment with low-dose stigmasterol and high-dose stigmasterol, the protein levels of PPAR*γ* and UCP-1 were significantly increased (*P* < 0.05), but the protein level of CPT-2 did not significantly change (*P* > 0.05) (Figure 4(b)). The immunofluorescence analysis showed that the fluorescence intensity of PPAR*γ* in group M was significantly reduced compared to that in group C (*P* < 0.05). After stigmasterol treatment, the fluorescence intensity of the PPAR*γ* protein was increased significantly (*P* < 0.05) (Figure 4(c)).

### 3.5. Generation of the PPAR*γ*-Knockdown Cell Line

**Figure 5:**
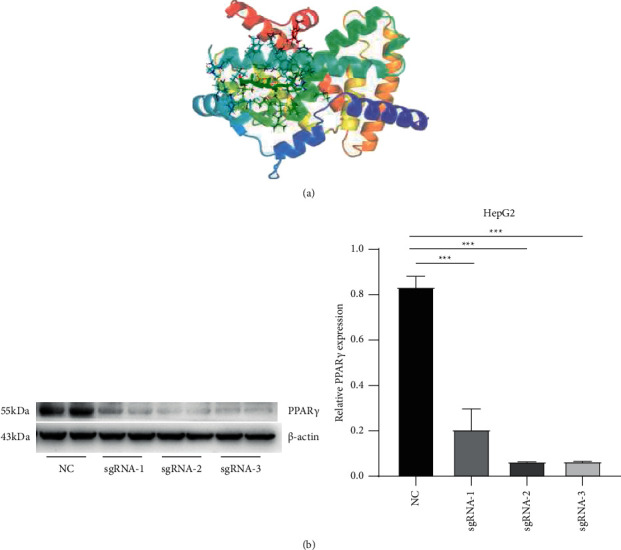
Generation of the PPAR*γ*-knockdown cell line. Knockdown cells were generated as described in the “Methods” section. (a) Schematic diagram of the docking activity of PPAR*γ* and stigmasterol. The binding free energy activity was −7.8 kcal/mol. (b) The PPAR*γ* gene in HepG2 cells was knocked down by transfecting a PPAR*γ* CRISPR/Cas9 plasmid. The transfected cells were screened with puromycin, and total cell protein was extracted for Western blot analysis to detect PPAR*γ* protein expression. ^*∗*^*P* < 0.05, ^*∗∗*^*P* < 0.01, and ^*∗∗∗*^*P* < 0.001 compared to the PPAR*γ*^−/-^ group. ^*∗*^*P* < 0.05, ^*∗∗*^*P* < 0.01, and ^*∗∗∗*^*P* < 0.001 compared to the PPAR*γ*^−/-^ group.

The results of docking stigmasterol with PPAR*α* and PPAR*γ* molecules were visualized with PyMOL software, and three-dimensional schematic diagrams of the binding of stigmasterol with PPAR*α* and PPAR*γ* were obtained. The best binding energies were −8.3 kcal/mol and −7.8 kcal/mol, showing that stigmasterol has good binding activity to both PPAR*α* and PPAR*γ* (Figure 5(a)).

Three cas9-gRNAs and one control empty vector were transfected into HepG2 cells, and puromycin was used for screening 48 hours after transfection. After all the untransfected normal cells died, the adherent cells of the transfection group were collected for WB experiments. The experimental results showed that the greatest knockdown of PPAR*γ* resulted from sgRNA3 transfection (Figure 5(b)). Thus, sgRNA3 was subsequently used to generate PPAR*γ*-knockdown cells for the drug intervention experiments.

### 3.6. Knockdown of PPAR*γ* Reverses the Effect of Stigmasterol on Blood Lipid Reduction

**Figure 6:**
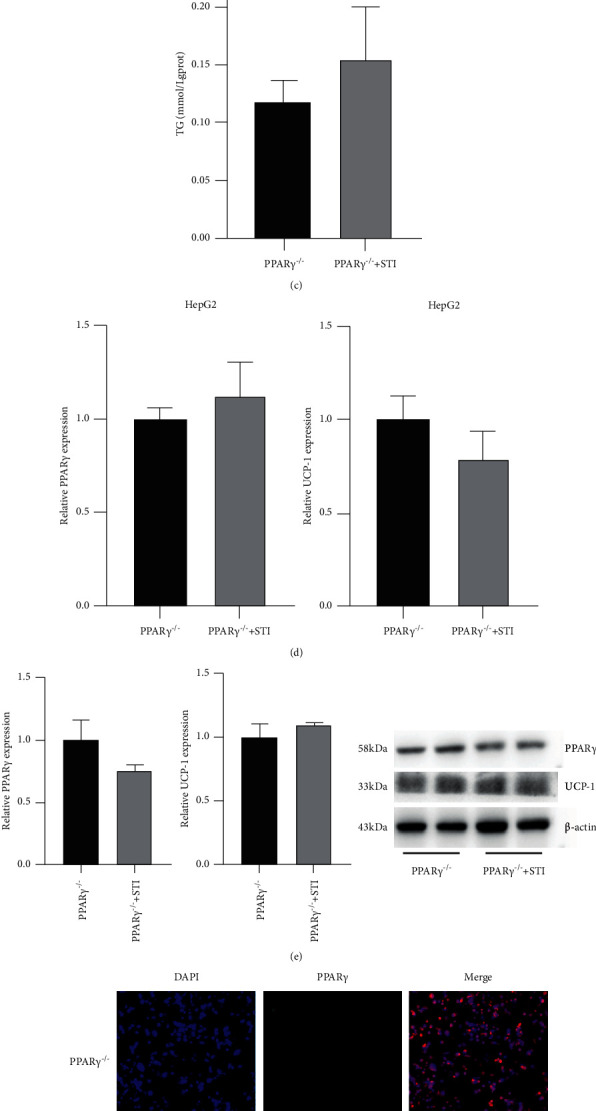
After knocking down PPAR*γ*, the lipid-lowering effect of stigmasterol was reversed. Treat the cells as described above. After the treatment, the cells were harvested. (a) Oil Red O experiment to detect the lipid accumulation level of cells in the PPAR*γ*^−/-^ group and PPAR*γ*^−/-^ + STI group. (b) Nile red stained, observed under a laser confocal microscope to obtain an image. (c) Detect the TG level by the kit and normalize it to the TG level. (d) RT-PCR to detect the mRNA expression level of PPAR*γ* and UCP-1. (e) Western blot detection of the protein expression level of PPAR*γ*, UCP-1. (f) Using immunofluorescence technology to detect the protein level of PPAR*γ*. Compared with the PPAR*γ*^−/-^ group, ^*∗*^*P* < 0.05 and ^*∗∗*^*P* < 0.01.

After stigmasterol treatment, Oil Red O staining showed that compared to the PPAR*γ*^−/-^ group, the PPAR*γ*^−/-^ + STI group showed no significant changes in the deposition of coloured lipid droplets, and there was no significant difference in the rate of lipid level change (Figure 6(a)). Nile red staining showed that compared to the PPAR*γ*^−/-^ group, the lipid content in the PPAR*γ*^−/-^ + STI group did not significantly change (Figure 6(b)). Compared to the PPAR*γ*^−/-^ group, the TG content in the PPAR*γ*^−/-^ + STI group did not significantly change (*P* > 0.05) (Figure 6(c)). The PCR analysis showed that compared to the PPAR*γ*^−/-^ group, the mRNA levels of PPAR*γ* and UCP-1 in the PPAR*γ*^−/-^ + STI group did not significantly change (*P* > 0.05) (Figure 6(d)). The WB analysis showed that compared to the PPAR*γ*^−/-^ group, the PPAR*γ*^−/-^ + STI histone levels of PPAR*γ* and UCP-1 did not significantly change (*P* > 0.05) (Figure 6(e)). The immunofluorescence analysis showed that compared to the PPAR*γ*^−/-^ group, the PPAR*γ* protein fluorescence intensity in the PPAR*γ*^−/-^ + STI group did not significantly change (*P* > 0.05) (Figure 6(f)).

## 4. Discussion

In recent years, researchers have studied the pharmacological properties of the Renshen and Chaihu herb pair in the treatment of metabolic diseases, especially NAFLD. Radix Bupleuri mainly plays a role in anti-inflammation, liver protection, and immune regulation [19–21]. Radix Ginseng is beneficial in improving glucose metabolism, lipid metabolism, liver function, and oxidative stress, and it has antifibrotic effects [22–24].

To investigate the pharmacodynamic mechanism of the Renshen and Chaihu herb pair in the treatment of metabolic diseases, our research group analysed the drug pair of the Renshen and Chaihu herb pair through network pharmacology and molecular docking technology. The results showed that stigmasterol is a common active ingredient in the Renshen and Chaihu herb pair, and the results suggested that PPAR*γ* may be a potential target. Importantly, antagonizing PPAR*γ* has previously been shown to attenuate NAFLD. Therefore, for this study, we hypothesized that stigmasterol, the active ingredient of the Renshen and Chaihu herb pair, may exert its therapeutic effect by activating PPAR.

The pathogenesis of NAFLD is complicated, and existing theories do not fully explain it. In the past, the “second hit” theory was the most popular explanation of NAFLD pathogenesis. Recently, an increasing number of possible mechanisms have been discovered that promote the development of NAFLD, including the production of lipotoxic lipids, the activation of inflammasomes, insulin resistance, intestinal flora imbalance, and hepatocyte fibrosis [25–29].

Therefore, to regulate glucose metabolism and lipid metabolism homeostasis, as well as to attenuate liver cell oxidative stress, improve mitochondrial energy metabolism, and reduce inflammation and fibrosis, researchers have sought a series of targets for the treatment of NAFLD. However, there are no specific drugs for NAFLD treatment. Insulin sensitizers, SGLT-2 inhibitors, lipid-lowering drugs, and antioxidants have shown certain effects in mitigating NAFLD, but there are also certain limitations to the use of these treatments, as revealed in previous studies: the insulin sensitizer, rosiglitazone, attenuates liver steatosis, hepatocyte inflammation, and fibrosis in patients with NASH [30]. Metformin improves measures of fasting blood glucose, insulin resistance, and serum adiponectin in NAFLD patients [31], but these results are controversial [32]. The SGLT-2 inhibitor, empagliflozin, reduces liver fat accumulation and lowers alanine aminotransferase levels in NAFLD patients [33]. Obeticholic acid, a selective FXR agonist, mitigates liver fibrosis in patients with NASH [34]. The antioxidant, vitamin E, attenuates liver steatosis, lobular inflammation, hepatocyte ballooning, and liver fibrosis in patients with NASH [35], but long-term use of vitamin E may increase the risk of stroke and prostate cancer [6]. Berberine reduces the levels of TG, TC, and LDL-c in patients with NAFLD, and it has a certain effect on liver function and blood sugar [36]. Pentoxifylline ameliorates liver steatosis, lobular inflammation, and fibrosis in patients with NASH [37]. Curcumin reduces the levels of alanine aminotransferase and aspartate aminotransferase by regulating the levels of total cholesterol and triglycerides to improve NAFLD [38]. Green cardamom has shown certain advantages in improving blood sugar and fatty liver in obese NAFLD patients [39].

Peroxisome proliferator-activated receptor (PPAR) belongs to the nuclear hormone receptor superfamily, which is divided into the following three subtypes: PPAR*α*, PPAR*β*/*δ*, and PPAR*γ*. PPAR*α* has been previously shown to play a role in regulating circulating lipids or cellular lipids by regulating liver and skeletal muscle lipid metabolism [40], and it is involved in peroxisome and mitochondrial *β*-oxidation, as well as FA transport and glucose production [41]. PPAR*β*/*δ* has been shown to participate in lipid oxidation and cell proliferation. A previous study showed that PPAR*γ* promotes adipocyte differentiation, and it is related to glucose metabolism and lipid uptake; PPAR*γ* also regulates the gene expression of glucose transporter 4 (GLUT4) and adiponectin (ADIPOQ) [42].

Downstream molecules of PPAR*γ* regulate the transcription of multiple genes. Pyruvate dehydrogenase kinase 4 (PDK4) is a member of the PDK/BCKDK protein kinase family, which inhibits the pyruvate dehydrogenase complex by phosphorylation of its subunits, and it also has been previously shown to participate in apoptosis and insulin resistance [43]. Acyl-CoA oxidase 1 (ACOX1) is involved in the fatty acid *β*-oxidation pathway. CD147 has been previously shown to inhibit fatty acid *β*-oxidation by activating the p38 MAPK signalling pathway, thereby downregulating PPAR*α* and its transcription target genes, namely, carnitine palmitoyltransferase 1A (CPT1A) and ACOX1 [44]. MaR1 also has been previously found to induce AMPK phosphorylation in DIO mice and primary hepatocytes, and AMPK inhibition completely blocks the effect of MaR1 on the expression of CPT1A, ACOX1, ATG5, and ATG7, providing a new treatment for ameliorating fatty liver [45]. Studies have also shown that LPIN2 and ST3GAL5 are new PPAR*β*/*δ* target genes and that the upregulation of PPAR*β*/*δ* gene expression is sensitive to plasma FFA levels [46]. Another study has shown that when CD36 decreases, adiponectin and uncoupling protein 2 (UCP2) increase [47]. Shenqi granules significantly reduce serum TC, TG, and LDL-C levels in a previous study, and they mediate FA uptake, affect mitochondrial energy metabolism, promote the tricarboxylic acid cycle, promote the transport of ATP from mitochondria to the cytoplasm, and restore mitochondrial function [48]. UCP-1 is also downstream of the three subtypes of PPAR*α*, PPAR*β*/*δ*, and PPAR*γ*, and it is highly expressed in brown adipose tissue. UCP-1 is a transmembrane channel protein located on the inner mitochondrial membrane, and it is the final effector molecule of brown adipose tissue in the process of heat production. For example, a previous study has suggested that after activation, UCP1 in the mitochondria that originally produced ATP is uncoupled and enters the mitochondrial matrix from the mitochondrial membrane space, and a large amount of heat is generated in the process, which is called adaptive thermogenesis [49]. After the sympathetic nervous system is stimulated by stress, protein kinase A was previously shown to activate downstream phosphorylation, promote the cysteine oxidation of UCP1, and activate its uncoupling properties to exert a thermogenic effect [50]. Therefore, activating UCP-1 has been suggested to exert a positive effect on weight loss, preventing hypertension and improving oxidative stress and other metabolic states [51]. When UCP-1 expression decreases, mitochondrial ROS increases, which may lead to insulin resistance in patients with type 2 diabetes.

In view of the multiple beneficial effects of PPAR in metabolism, seeking PPAR agonists has always been a hot research topic in NAFLD. However, the clinically used PPAR*α* agonists, fibrates, have not shown a significant improvement in NAFLD. In addition, a previously performed meta-analysis has shown that the PPAR*γ* agonist pioglitazone has a beneficial effect on advanced fibrosis in patients with NASH [52]. Pioglitazone can improve liver steatosis, lobular inflammation, and balloon dilatation, but its effect on weight gain and its inability to increase the improvement rate of NAFLD limit its further development. Therefore, an increasing number of researchers are devoted to the development of dual or multiple agonists of PPAR. Studies have shown that saroglitazar, a dual agonist of PPAR*α*/*γ*, improves insulin resistance and steatohepatitis in NASH mice [53]. Studies have also reported that the PPAR pan-agonist can reduce steatosis, reverse liver injury and monocyte infiltration, and reduce the inflammatory response in macrophages and monocytes with the participation of PPAR*δ* [54].

Phytosterols are widely distributed in a variety of plant cells, including vegetable oils, plant seeds, and pollen, and they function in the maintenance of the stability of plant cell membranes [55]. More than 200 different plant sterols have now been identified. Phytosterols usually exist in two different forms: one form is in a free state, and the other is covalently bound to ester or glycosidic bonds. Recent studies have found that plant sterols regulate cholesterol metabolism-related proteins to reduce circulating cholesterol levels [56]. It helps alleviate the risk of lipid metabolism disorders, cardiovascular disease, diabetes, and tumours [57–60]. In addition, the intervention of plant sterols also has certain advantages in increasing the abundance of intestinal flora, regulating faecal metabolites, and ameliorating NAFLD [61]. The most common phytosterols are *β*-sitosterol, campesterol, and stigmasterol.

In the past, many studies have reported the therapeutic effects of stigmasterol in antioxidative activities, anti-inflammatory activities, regulation of lipid metabolism, and antitumour activities. Studies have shown that stigmasterol improves rat brain damage caused by ischaemia-reperfusion by reducing oxidative stress and inflammation [62]. Stigmasterol relieves collagen-induced arthritis in rats by inhibiting proinflammatory cytokines [63]. In mice, stigmasterol significantly reduces total lipid, and TG and TC levels in the liver as well as liver histopathological levels, and it reduces intestinal bile acid levels and increases faecal lipid levels to treat NAFLD [64]. Stigmasterol activates ROS production and calcium overload, and it induces mitochondrial apoptosis and inhibits tumour cell migration and angiogenesis [65]. It has also been reported that stigmasterol inhibits the JAK/STAT signalling pathway and blocks the cell cycle to exhibit certain antitumour effects [66]. Stigmasterol has shown certain potential for the treatment of metabolic diseases, but there is no report that stigmasterol can treat metabolic diseases through PPAR*γ*.

A safety test of WST-1 was performed in the current study, and we found that stigmasterol at less than 1000 *μ*M was not significantly toxic to HepG2 cells. We also used free fatty acids in a high-fat cell model treated with different doses of stigmasterol. The stigmasterol treatment had the following effects in WT cells: intracellular pigmented lipid droplets were deposited; the lipid content was significantly decreased; the TG content was significantly decreased; the PPAR*γ*, CPT-2 and UCP-1 mRNA levels were significantly increased; and the PPAR*γ* and UCP-1 protein levels were significantly increased. Subsequently, our group generated a HepG2 cell line in which PPAR*γ* was knocked down. Stigmasterol treatment in these PPAR*γ*^−/-^ cells showed the following results: there was no significant change in the deposition of coloured lipid droplets; there was no significant difference in the lipid content; there was no significant difference in TG content; and the PPAR*γ* and UCP-1 mRNA and protein levels did not significantly change.

In this study, the main active ingredients of the Renshen and Chaihu herb pair were discovered using a network database system. In vitro experiments demonstrated that stigmasterol enhances the lipid metabolism of HepG2 cells through the PPAR*γ*-UCP-1 signalling pathway. In recent years, an increasing number of studies have focused on the discovery of PPAR dual agonists and even pan-agonists for the treatment of NAFLD. This study further explored the effect of stigmasterol on PPAR*α* and PPAR*δ*, demonstrating its potential mechanism in the treatment of NAFLD.

## Figures and Tables

**Table 1:** Basic information on the 17 active components in Chaihu.

Molecular ID	Name	OB	DL
MOL001645	Linoleyl acetate	42.1	0.2
MOL002776	Baicalin	40.12	0.75
MOL000449	Stigmasterol	43.83	0.76
MOL000354	Isorhamnetin	49.6	0.31
MOL000422	Kaempferol	41.88	0.24
MOL004598	3,5,6,7-Tetramethoxy-2-(3,4,5-trimethoxyphenyl) chromone	31.97	0.59
MOL004609	Areapillin	48.96	0.41
MOL013187	Cubebin	57.13	0.64
MOL004624	Longikaurin A	47.72	0.53
MOL004628	Octalupine	47.82	0.28
MOL004644	Sainfuran	79.91	0.23
MOL004648	Troxerutin	31.6	0.28
MOL004653	(+)-Anomalin	46.06	0.66
MOL004702	Saikosaponin c_qt	30.5	0.63
MOL004718	*α*-Spinasterol	42.98	0.76
MOL000490	Petunidin	30.05	0.31
**MOL000098**	**Quercetin**	**46.43**	**0.28**

**Table 2:** Basic information on the 22 active components in Renshen.

Molecular ID	Name	OB	DL
MOL002879	Diop	43.59	0.39
MOL000449	Stigmasterol	43.83	0.76
MOL000358	*β*-Sitosterol	36.91	0.75
MOL003648	Inermin	65.83	0.54
MOL000422	Kaempferol	41.88	0.24
MOL004492	Chrysanthemaxanthin	38.72	0.58
MOL005308	Aposiopolamine	66.65	0.22
MOL005314	Celabenzine	101.88	0.49
MOL005317	Deoxyharringtonine	39.27	0.81
MOL005318	Dianthramine	40.45	0.2
MOL005320	Arachidonate	45.57	0.2
MOL005321	Frutinone A	65.9	0.34
MOL005344	Ginsenoside rh2	36.32	0.56
MOL005348	Ginsenoside-Rh4_qt	31.11	0.78
MOL005356	Girinimbin	61.22	0.31
MOL005357	Gomisin B	31.99	0.83
MOL005360	Malkangunin	57.71	0.63
MOL005376	Panaxadiol	33.09	0.79
MOL005384	Suchilactone	57.52	0.56
MOL005399	Alexandrine_qt	36.91	0.75
MOL005401	Ginsenoside Rg5_qt	39.56	0.79
**MOL000787**	**Fumarine**	**59.26**	**0.83**

**Table 3:** Results of docking Renshen and Chaihu herb pair components with core targets.

Target	Renshen and Chaihu herb pair components	Highest binding energy (kcal/mol)
AKT1	Girinimbin	−10.2
AKT1	Quercetin	−8.8
AKT1	Kaempferol	−8.6
AKT1	Petunidin	−8.6
AKT1	Areapillin	−8.5
AKT1	Isorhamnetin	−8.5
AKT1	3,5,6,7-Tetramethoxy-2-(3,4,5-trimethoxyphenyl) chromone	−8.4
MAPK3	*α*-Spinasterol	−9.2
PPARA	Stigmasterol	−8.3
PPARA	Aposiopolamine	−7.2
PPARA	Arachidonate	−7.2
PPARA	*α*-Spinasterol	−7
PPARG	*α*-Spinasterol	−8.1
PPARG	Stigmasterol	−7.8
PPARG	*β*-Sitosterol	−7.6
PPARG	Arachidonate	−6.6
PPARG	Ginsenoside Rh4 qt	−6.4
SIRT1	Fumarine	−8.4
SIRT1	Sainfuran	−7.6
STAT3	Ginsenoside rh2	−6.9
STAT3	Saikosaponin cqt	−6.8
STAT3	Ginsenoside Rg5 qt	−6.6
STAT3	Ginsenoside Rh4 qt	−6
STAT3	Alexandrine qt	−5.5
TNF	Baicalin	−7.6
**TP53**	**Arachidonate**	**0**

## Data Availability

The dataset generated from this study can be requested from the corresponding author (Prof. Bing Wang) for scientific purposes.

## Supplementary Materials

Supplementary Materials There are 2 supplementary tables in the submitted supplementary materials. Supplemental Table 1: GO enrichment analysis of the intersection target of the herb pair and NAFLD. Supplemental Table 2: KEGG pathway enrichment analysis of the intersection target of the herb pair and NAFLD.
