# Supplementary material for: A Network Pharmacology Study on the Active Components and Targets of the Radix Ginseng and Radix Bupleuri Herb Pair for Treating Nonalcoholic Fatty Liver Disease
Source: Evid Based Complement Alternat Med. 2022 Feb 8;2022:1638740. doi: 10.1155/2022/1638740 (PMC8846978; doi:10.1155/2022/1638740)
Supplement: Supplementary Materials — There are 2 supplementary tables in the submitted supplementary materials. Supplemental Table 1: GO enrichment analysis of the intersection target of the herb pair and NAFLD. Supplemental Table 2: KEGG pathway enrichment analysis of the intersection target of the herb pair and NAFLD. [file 1638740.f1.docx]

Supplemental Table 1 GO enrichment analysis of the intersection target of herb pair and NAFLD

| Description | Type | Number of genes | P-value | P-adjust |
| --- | --- | --- | --- | --- |
| Regulation of small molecule metabolic process | Biological process | 24 | 8.47E-23 | 2.76E-19 |
| Regulation of lipid metabolic process | Biological process | 22 | 4.33E-21 | 7.05E-18 |
| Positive regulation of small molecule metabolic process | Biological process | 15 | 2.18E-18 | 2.37E-15 |
| Positive regulation of lipid metabolic process | Biological process | 14 | 4.56E-17 | 3.72E-14 |
| Steroid metabolic process | Biological process | 17 | 5.43E-16 | 3.54E-13 |
| Lipid localization | Biological process | 18 | 6.63E-16 | 3.60E-13 |
| Transcription initiation from RNA polymerase II promoter | Biological process | 14 | 1.64E-15 | 7.65E-13 |
| Lipid transport | Biological process | 17 | 2.75E-15 | 1.12E-12 |
| RNA polymerase II transcription factor complex | Cellular component | 9 | 2.33E-09 | 4.69E-07 |
| Nuclear transcription factor complex | Cellular component | 9 | 1.46E-08 | 1.47E-06 |
| Membrane raft | Cellular component | 10 | 5.68E-08 | 2.94E-06 |
| Membrane microdomain | Cellular component | 10 | 5.85E-08 | 2.94E-06 |
| Membrane region | Cellular component | 10 | 8.28E-08 | 3.33E-06 |
| Transcription factor complex | Cellular component | 9 | 2.27E-06 | 7.60E-05 |
| Neuronal cell body | Cellular component | 9 | 2.70E-05 | 0.000775 |
| Apical part of cell | Cellular component | 7 | 0.000218 | 0.005474 |
| Steroid hormone receptor activity | Molecular function | 11 | 6.10E-17 | 1.91E-14 |
| Monocarboxylic acid binding | Molecular function | 10 | 2.02E-14 | 3.16E-12 |
| Nuclear receptor activity | Molecular function | 9 | 6.20E-14 | 4.85E-12 |
| Transcription factor activity, direct ligand regulated sequence-specific DNA binding | Molecular function | 9 | 6.20E-14 | 4.85E-12 |
| Fatty acid binding | Molecular function | 8 | 2.75E-13 | 1.72E-11 |
| Carboxylic acid binding | Molecular function | 10 | 1.46E-09 | 7.61E-08 |
| Organic acid binding | Molecular function | 10 | 2.62E-09 | 1.17E-07 |
| Nuclear receptor transcription coactivator activity | Molecular function | 7 | 3.78E-09 | 1.48E-07 |

Supplemental Table 2 KEGG pathway enrichment analysis of the intersection target of herb pair and NAFLD

| Description | Number of genes | P-value |
| --- | --- | --- |
| Insulin resistance | 13 | 2.60E-13 |
| Adipocytokine signaling pathway | 9 | 8.13E-10 |
| PPAR signaling pathway | 9 | 2.22E-09 |
| AMPK signaling pathway | 9 | 1.14E-07 |
| Longevity regulating pathway | 8 | 1.51E-07 |
| FoxO signaling pathway | 9 | 2.44E-07 |
| AGE-RAGE signaling pathway in diabetic complications | 8 | 3.76E-07 |
| Insulin signaling pathway | 9 | 4.06E-07 |
| Prolactin signaling pathway | 7 | 4.62E-07 |
| Type II diabetes mellitus | 6 | 6.57E-07 |
| Non-alcoholic fatty liver disease (NAFLD) | 9 | 7.32E-07 |
| Longevity regulating pathway - multiple species | 6 | 3.96E-06 |
| Gastric cancer | 8 | 7.73E-06 |
| Glucagon signaling pathway | 7 | 7.75E-06 |
| Hepatitis C | 8 | 1.03E-05 |
| Toxoplasmosis | 7 | 1.12E-05 |
| Pancreatic cancer | 6 | 1.31E-05 |
| EGFR tyrosine kinase inhibitor resistance | 6 | 1.63E-05 |
| Hepatocellular carcinoma | 8 | 1.86E-05 |
| Colorectal cancer | 6 | 2.66E-05 |
